# Experiences of policy-advisors in capacity, opportunity and motivation for implementing local tobacco control

**DOI:** 10.1186/s12889-024-21099-z

**Published:** 2024-12-23

**Authors:** Sophie J.A. Jooren, Gemma Geuke, Marc C. Willemsen, Maria W. J. Jansen, Jeroen Bommelé

**Affiliations:** 1 Department of Health Promotion, School of Public Health and Primary Care (CAPHRI), Maastricht University, Maastricht, The Netherlands; 2 The Netherlands Expertise Centre for Tobacco Control, Trimbos Institute, Utrecht, The Netherlands; 3 Department of Health Services Research, School of Public Health and Primary Care (CAPHRI), Maastricht University, Maastricht, The Netherlands

**Keywords:** Smoking, Municipalities, Local tobacco control, Smoke free policy

## Abstract

**Introduction:**

Due to decades of decentralisation of public health policies, local governments have been given increased tobacco control responsibilities across European countries. Previous studies suggest that implementing local tobacco control policies is not without challenges (e.g., lack of resources and enforcement capabilities). This study investigates the policy implementation of both smoke-free environments and smoking cessation support services by local public health professionals in the Netherlands.

**Method:**

We interviewed 24 officials of regional public health departments about the barriers and facilitators of implementing local tobacco control policies. Interviews were transcribed, analysed and classified using the Behaviour Change Wheel model (COM-B) comprising of the three components capability, opportunity, and motivation.

**Results:**

Personal motivation was an important condition for public health professionals to work on specific subtopics within tobacco control. Smoke-free environments were generally considered most motivating to work on, but also involve practical obstacles such as a lack of enforcement capabilities. Smoking cessation support services were reported to be less attractive to work on, as there are no clear guidelines on what public health professionals could and should do regarding smoking cessation support.

**Conclusion:**

Municipalities and local public health departments may contribute to tobacco control by creating smoke-free areas and offering services to help people stop smoking. The national government of the Netherlands could support local governments by providing clearer guidelines on creating smoke-free spaces and on how to improve local smoking cessation support services.

## Introduction

More than eight million people die each year from tobacco use or from being exposed to tobacco smoke [1]. Governments can play a large role in preventing these deaths and have done so in the past by implementing a wide range of tobacco control measures. Despite this progress, the WHO still underscores the importance for governments to prioritize tobacco control [2]. Due to decades of decentralisation of public health policy across Europe, local governments are given an increased role in multiple public health tasks across European countries, including tobacco control [3]. For example, the national government of the Netherlands has decentralised health promotion as this was deemed to improve operational efficiency [4], and municipalities are expected to contribute to national tobacco reduction goals. The WHO acknowledges the role of local governments in helping to protect people from tobacco smoke exposure [2].

Previous studies, for example in Australia and California, identified opportunities for local tobacco control policy [5–7]. Mark et al. [5] showed that, since introducing smoke-free policies in Australia, the proportion of local councils with tobacco control policies has increased from 18 to 64%. However, previous studies also report that implementing local tobacco policies is challenging. These challenges include insufficient staff and resources, difficulties with enforcing tobacco control policies, bureaucracy, and lack of support from local councils [5–7]. Studies in Europe often focus on the role of local organisations, rather than the role of municipalities.

### COM-B model

To study behavioural facilitators and barriers of policy implementation, several models can be used. One of them is the behaviour change wheel (COM-B) [8]. In this model, capability, opportunity, and motivation (COM) are considered the three main interrelated determinants of the behaviour (B) of policy-advisors. *Capability* is an individual’s psychological and physical capability to engage in an activity. This includes having the necessary skills to participate in a certain activity or policy implementation process. *Opportunity* refers to the physical and social environment, such as the prevalence of smoking-or tobacco related environmental cues and the predominant communication discourse in society about smoking. *Motivation* entails reflective motivation (reflective decision-making processes) and automatic motivation (emotion and impulses). It encompasses routine processes, emotional responses, and analytical decision-making. An example of automatic motivation is resistance to change. This could be the resistance to implement policies targeting individual support in smoking cessation regarding smoking by a policy-advisor who believes smoking is an individual choice. In this study, we distinguish the COM components for the sake of clarity and readability, but ultimately, behaviour is determined by an interaction of all components.

In this study, we apply the COM-B model to study the determinants of the organizational behaviour of public health policy-advisors in implementing local tobacco control. We do this in line with previous research which investigated organizational behaviours of local policy-makers at the strategic, tactical and operational levels on the subject of childhood obesity [9]. We use the COM-B model to identify the main determinants of behaviour of policy-advisors. This could inform policymakers of develop strategies to make decentralized tobacco control policies more effective.

### Public health departments in the Netherlands

In the Netherlands, tobacco control is relatively well developed. A nationwide smoking ban has been applied and enforced in all indoor public spaces, workplaces, including the hospitality sector. In addition, all outdoor school premises are legally designated as smoke-free zones [10, 11]. However, while the law allows smoking in other outdoor areas, promoting voluntary smoke-free outdoor areas is an important responsibility for municipalities. For smoking cessation care, reimbursements are determined by the healthcare insurance providers [12]. Public health departments can contribute to smoking cessation care by promoting this among the entire population and coordinating the different partners involved in the region [13]. In general, municipalities work on public health by developing public health policies, ensuring health protection, health promotion, and monitoring overall health trends [14]. These responsibilities align with a broader trend of decentralization in Europe, where local governments are increasingly entrusted with greater responsibilities [15]. However, these responsibilities often do not include a legislative power. Unlike local governments in the United States—on which much of the international literature on local tobacco control is focused—European municipalities often lack the authority to legally implement and enforce necessary tobacco control measures that help contribute to national governmental goals.

In 2018, the national government of the Netherlands committed to financially support municipalities in developing local Smoke-Free Generation policies as part of a National Prevention Agreement [16]. One of these financial support instruments is a grant for regionally-based public health departments. Public health departments are joint arrangements of municipalities and are governed by the aldermen of the participating municipalities. In total, there are 25 public health departments in the Netherlands, each responsible for a number of municipalities ranging from 6 to 26 with 600.000 to 800.000 inhabitants.

Public health departments were invited to apply for a grant that would enable them to support local governments in the region with tobacco control activities. This grant totalled €80.000 per region and was made available for the period 2018–2021. A follow-up grant was distributed for 2023–2025. The grant prioritized initiatives aimed at embedding smoke-free policies within public health regulations and prevention agreements, creating smoke-free environments, addressing the needs of low socioeconomic status groups, and promoting effective and accessible smoking cessation care [13, 17]. In a previous study, we critically reviewed the content of these grant proposals [13].

### Research aim

Insight in the behavioural barriers and facilitators of the policy implementation process on local tobacco control is currently lacking. We therefore set out to examine behavioural barriers and facilitators encountered by public health policy-advisors regarding (1) their role in creating smoke-free outdoor areas and (2) their contribution to providing smoking cessation support. These topics are the two major tobacco control policies issues for which municipalities in the Netherlands have a role [13]. We also investigate which supportive instruments are already in place for public health professionals to support the policy implementation process. Finally, we investigate which instruments may enable different behaviours among these professionals.

## Methods

### Context, sampling strategy and data collection

In March, April, and May 2022, we invited grant project leaders of all 25 public health departments to participate in an interview about their experience in implementing tobacco control policies. Contact information of project leaders was publicly available through the Smoke-free Generation website [18]. The invitation was sent by email, including an information letter on our research. A maximum of two reminders were sent if respondents did not reply. If respondents replied positively, we sent them a consent form, an information letter on our data management approach, and scheduling options for the interview. Due to the coronavirus restrictions, respondents could choose between an online or an in-person interview. We only conducted in-person interviews if they were compliant with the prevailing Covid-19 pandemic regulations at that time. Twenty-four out of twenty-five local project leaders replied, made an appointment, and consented to conduct an interview. One project leader was unable to participate due to time constraints. Interviews were conducted between April 2022 and July 2022 and lasted 51–75 min. All interviewees signed the consent form. All respondents worked as a public health professional at the public health department. We will refer to them in this paper as public health professionals from this point on. Seventeen interviews were held online and seven in-person.

### Qualitative approach and data analysis

After conducting twenty-four interviews, we achieved data saturation. Interviews were transcribed verbatim and analysed using MAXQDA [19]. We analysed according to open and axial coding, to keep an open mind for the data [20]. The first and second author analysed both interviews separately and discussed their codebooks after five, ten, and fifteen interviews, and again after the remaining nine interviews. No major discrepancies were found. Similar codes were put into overarching codes. Within the thematic analysis, these overarching codes were classified following the COM-B model and the coders established agreements on the most important themes in each of the components. The results section was structured accordingly Both coders first coded separately and then discussed any discrepancies in the classification.

### Member check

We organised a member check to ensure that data was interpreted correctly, that interviewees’ stories were accurately represented, and to address gaps in the available data [21]. In March 2023, all interviewees were invited to an in-person group meeting. Fourteen out of twenty-four public health professionals attended. The meeting began with the first author presenting interview results, seeking agreement from attendees via direct inquiry and a mobile-based feedback tool. Attendees were also able to ask questions during the presentation. The second part involved discussions around four topics (evaluation, role of public health professionals, budget, and smoking cessation support services), chosen for allowing for diverse viewpoints, with attendees choosing statements of interest for further discussion. Subsequently, all topics were collectively discussed. The meeting concluded with an open exchange among participants. This part of the meeting was led by GGD GHOR Nederland, the umbrella organisation for public health departments in the Netherlands.

### Ethical approval

Ethical approval was received by the ethics review committee of the Faculty of Health, Medicine and Life Sciences of the Maastricht University (FHML-REC/2022/021). All participants provided written consent for the interview and granted permission for the anonymized use of their data in publication.

## Results

We will describe the results according the COM-B model and the corresponding instruments below.

### Creating smoke-free environments

*Capability.* Public health professionals expressed that flexibility was an important skill in creating smoke-free environments, as many local parties are involved in this creation process, such as schools, sports clubs, playgrounds, and various departments of the local municipalities. Processes and opinions often differed between these parties, meaning that the officials were frequently involved in discussions on the practical implementation of these environments:

*“Someone who focuses more on that environment and spatial planning*

*immediately thinks of questions such as: how are we going to enforce that*

*and how can these [smoke-free] signs be put up? You notice that those differences in*

*vision are still there.” (Interview 005)*.

Public health professionals also had to organise a diverse set of resources simultaneously: manpower, time, money, and local officials that took up the lead on this theme. Furthermore, the size of a municipality appears to affect the implementation of smoke-free environments. Larger municipalities had more capacity, but also more bureaucracy. Interviewees experienced closer connections with local stakeholders in smaller municipalities, meaning that, while less resources were available, implementation also required less resources.

*Opportunity*. The public health professionals encountered various practical obstacles to creating smoke-free environments, including nuisance from smokers in residential areas as they moved away from the now smoke-free environments or dealing with the waste of cigarette butts due to the removal of ashtrays. Additionally, public health professionals were unsure if enforcement was their responsibility and how enforcement should take place, as smoke-free environments are not formally included in the national law. They wondered whether the focus should be on enforcement or norm change. Therefore, they considered whether a tailored local approach would be more useful or the inclusion of smoke-free environments in the law. One public health professional, for instance, told about a sports club that wanted to wait until it was included in the law:

*“There are sports clubs that say well*,* you know*,* we just*

*had corona*,* everyone starts to come back. Then I start a conversation*

*about smoke-free and I will lose my volunteers*,* […] we will wait until*

*it is included in national law that we need to be smoke-free.” (Interview 011)*.

*Motivation.* Creating smoke-free environments is motivating, as public health professionals found the environments essential to protect children from tobacco smoke and that smoke-free environments conveyed a positive message. They also believed they could contribute to a change in non-smoking norms and said they could reach a broad target group through smoke-free environments. Demotivating factors were concerns about the effectiveness of smoke-free environments, especially placing smoke-free signs and street tiles without providing any additional information:

*“I can’t think of a municipality that has also provided extensive information*

*to playground owners or swimming pool owners. It was really just placing*

*signs and checking off. […] Simply placing signs is of course not very effective*,

*but I think*,* you know*,* it is one of the many interventions you do.’’ (Interview 008)*.

Another less motivating factor was monitoring the number of new smoke-free environments. It was a significant task for public health professionals to keep track of all smoke-free environments, and thus to really see their progress:

*“Then I ask all my colleagues*,* what is the situation in your municipality*,

*what steps have been taken? […] let me know if there is a new sports club*

*or a new place that is smoke-free*,* and I will put it on the list. That is difficult.*

*I can’t guarantee I’ll have all smoke-free locations*,* that’s just very difficult.” (Interview 003)*.

*Instruments to COM-B.* Interviewees suggested four instruments by which local implementation could be improved. Firstly, they mentioned smoke-free outdoor environments could be included in national law. Some smoke-free outdoor environments, such as schools, are already included in the law, while other smoke-free outdoor areas, such as playgrounds, were regulated locally. A second instrument to ease cooperation with local parties was that smoke-free signs could be provided for free by national government. Thirdly, public health professionals felt the need for detailed guidelines on implementing local policies. For instance, some public health departments strived for smoke-free outdoor places in areas frequented by children, but which were also for all age groups, such as (outside) workplaces for adults. Public health professionals were unsure how to effectively manage these different interests. A fourth instrument entailed communication. Interviewees found it important that a nationwide platform to track the number of smoke-free environments would be made available. Also, it would be helpful if only one single website about local implementation of smoke-free environments existed to prevent fragmentation of information.

### Developing smoking cessation support services

*Capability.* Public health professionals highlight insufficient resources, time, capacity, and competencies as key barriers hindering them from actively implementing support services. Moreover, one public health professional advocated for a more practical approach, acknowledging the differences in skills and the level of training among personnel in various public health services, which hindered their ability to provide individualized addiction care.

*“Then you must be able to choose to work with one municipality*,* because*

*then you really have to appoint someone who can be a helping hand in the neighbourhood. […] So if we wanted to do that*,* we would have to hire*

*someone else who could do that*,* who has those competencies.” (Interview 002)*.

Public health professionals also argued that (health care) professionals must be able to engage in effective communication regarding smoking cessation, encompassing not only patients but also other relevant target groups, including parents. Making sure everyone has the right discussion tools for these conversations is key here.

*“I think it has been a very nice sequence*,* and that now the time is more ripe*

*to talk about quitting. Because you also notice that in organizations*,* they make*

*it smoke-free*,* but you do get to have a conversation with your smokers. So we sometimes get those questions*,* how should we do that?” (Interview 019)*.

*Opportunity.* On the one hand, public health professionals noted that increasing the number of smoke-free environments paved the way for increasing smoking cessation support. They argued that you can simply start by making smoking cessation support services more accessible, strengthening the local network for smoking cessation support services, and advocating for smoking cessation in health care (such as maternity care).

*“We also try to make a lot of effort to ensure that smoking cessation help is*

*also made known*,* so what kind of help is available in our region? And I recently*

*made an overview of that and we are now going to discuss how can we best*

*distribute it.” (interview 015)*.

On the other hand, some public health professionals were hesitant to work on smoking cessation support services. Their arguments are closely related to the lack of capability and motivation: they did not perceive this as a suitable task for the public health departments (as it only targeted smokers, rather than the general public), did not know where to start, or simply did not see how sufficient resources could be allocated to effectively manage this.

Public health professionals also addressed that finances have an important role for this issue. For the public health departments, short-term financing was a first good step, especially for projects such as training healthcare providers or improving findability within healthcare networks. But in order to really provide care or to really strengthen and expand local smoking cessation networks, structural financing was needed.

*“We’ll leave the whole smoking cessation support services for now […]*.

*this is a temporary project with extra hours*,* but you actually want something*

*to remain there afterwards. And I have more hope for this with smoke-free*

*environments than with information or other matters that remain dependent*

*on people and money. I assume that once a playground is smoke-free*,* it will*

*remain so.” (interview 006)*.

Additionally, for smokers, public health professionals felt that the national government should offer more opportunity to quit smoking. Currently, smokers are only reimbursed for one quit attempt per year by their health insurance company.

*“But these are all things that should change nationally. Or there should be*

*another offer*,* or whatever*,* more compensation. And that is sometimes difficult*,* because you actually need the government to achieve your local goals.”*

*(interview 001)*

*Motivation.* The motivation of public health professionals closely depended on the opportunities they saw for this topic. On the one hand, interviewees mentioned concrete goals that they considered suitable for the local level and made them feel motivated. On the other hand, public health professionals were less motivated to work on the topic as they did not perceive this as a suitable task for the public health departments.

*Instruments to COM-B.* Public health professionals addressed several instruments to improve implementation of smoking cessation services. Firstly, guidelines from the national government could reduce ambiguity on what role is expected of the local authority in smoking cessation support. One interviewee noted that the national government and other organisations often suggested starting smoking cessation support projects in specific neighbourhoods, while they considered the resources for such a public health service simply insufficient:

*“I sometimes wonder whether other parties understand how a public health service works*,* how it is organized and what kind of people work here. […] We are responsible for all municipalities and the whole municipality and not for one district within one municipality.” (interview 002)*.

Secondly, public health professionals preferred more collaboration and training with the national government about the coordination of the national smoking cessation care campaigns to embed them on the local level or give them a local twist.

*“And we have tried to make the national campaign a bit more local […] As a public health department you can*,* it is just difficult to really to set up entire campaigns. And I think the [national] campaigns now*,* yes*,* they don’t really pop off for me. […] And perhaps we should be more facilitated in this by such institutions*,* how can you give that a local twist?” (interview 007)*.

## Discussion

We explored behavioural barriers and facilitators encountered by public health professionals implementing smoke-free outdoor areas and improving local smoking cessation support services. Within the COM-B model, we identified several major themes. In the upcoming section, we will discuss key challenges of creating smokefree areas. We propose solutions by focusing on three main aspects: the adaptability of public health professionals, their motivation, and the lack of a clearly defined formal or mandated role. Similarly, in the second part, we will discuss whether smoking cessation support services are appropriately designated as a local-level responsibility. We also explore regional variations and discuss the nature of collaborations in this area.

### Smoke-free environments

We found that public health professionals could coordinate a diverse set of recourses simultaneously to create smoke-free environments: manpower, time, money, and involving different parties such as schools, sports clubs, and playgrounds. This required flexibility in time management and the ability to shift focus frequently. As part of the implementation of smoke-free environments lies with local parties, public health professionals and municipal officials rely on local organizations and community cooperation for the implementation of outdoor smoke-free environments. This is particularly true for environments other than schools, as they are not formally included in the national smoke-free law. Additionally, besides public health, different departments within the municipality were often involved, such as public space or safety, and differing opinions among them led to frequent discussions about the practical implementation of smoke-free environments.

Overall, public health professionals were quite well-motivated to work on smoke-free environments. Even municipalities that were previously hesitant to adopt tobacco control policies, now actively distributed smoke-free signs. However, some expressed doubts on whether only placing smoke-free signs helps to truly make an environment smoke-free and ensure that people do not smoke. While such scepticism does not tend to deter public health departments from engaging with smoke-free activities, it could impact how much effort they invest. We found that motivated professionals actively implement as much different smoke-free environments as possible, while those less motivated limit their efforts to the basic task of providing local organisations with no-smoking signs. As a consequence of these individual differences and preferences, we see large variations between public health departments. Some municipalities have many designated smoke-free environments and people feel free to confront smokers if they do not adhere to the smoking restrictions. In contrast, in other areas, there is only a limited number of smoke-free outdoor environments and smoking is less denormalised.

The absence of a clear formal role, and uncertainty about the emphasis on enforcement, influence the implementation of smoke-free environments. Firstly, the absence of a clear formal role regarding how to contribute to national or local tobacco control goals, negatively impacts the public health professional’s motivation to work on outdoor smoke-free environments. The health professional’s work would thus benefit from clear national guidelines. For example, if the percentage of (young) smokers exceeds a certain limit or the national average, the municipality is required to take additional measures regarding smoke-free areas and smoking cessation support services. For other themes, such target values are quite common. Within environmental legislation, for example, the concentration of numerous substances in the air, groundwater, sewage, soil, and many other places must be closely monitored and compared against precisely determined intervention values [22, 23]. Other studies also recommended this. For instance, a study in the United Kingdom looked at perceptions of how useful guidelines are for local governments [24]. It showed that the absence of specific recommendations from the national government hindered effective communication. Such recommendation could, for instance, be included in the grant for public health departments of the national government.

Secondly, the implementation of smoke-free environments is difficult if there is uncertainty about whether local policymakers are mandated to enforce smoke-free legislation. The national government could engage with public health departments and municipalities to determine how national legislation may facilitate local enforcement of smoke-free environments and decide on which level of governance is responsible for enforcement. It is important to consider the desirability of local enforcement as part of implementing national smoking bans. Public health professionals in this study suggested that enforcement should not be the primary focus of implementing smoke-free environments. They recommended that, instead, local governments could prioritise fostering behavioural and normative change by making many environments smoke-free, educating local parties about the importance of smoke-free environments, and ensuring the development of a culture where people feel comfortable addressing each other. As Wynne et al. [25] also show, policy awareness is most important when implementing smoke-free outdoor environments. National smoke-free laws could help public health professionals to create smoke-free spaces, but enforcement should not be the primary aim at the local level.

### Smoking cessation support services

Public health professionals expressed that they saw little possibility to improve local smoking cessation support services, as they did not perceive this as their designated task. Their focus is on population-based prevention measures such as smoke-free environments. While some public health professionals expressed ideas about what they could do on this topic, others struggled with the complexity of smoking cessation support services, which in the Netherlands has been privatised. Some public health departments find it difficult to coordinate the many stakeholders involved in smoking cessation support services within their region, such as general practitioners, mental health professionals, and youth care workers. Uncertainty about tasks or difficulty in taking them on can lead to inconsistent efforts across regions, with some regions actively engaged and others less, depending on the person shaping the policy.

We noted differences between public health departments concerning the implementation of smoking cessation support services. In one region, smokers received assistance to quit smoking, because the public health department has trained its employees to become quit-smoking coaches. However, in other regions, this support may be lacking due to insufficient capability or motivation among public health professionals. The national government could clarify the role of public health departments in the smoking cessation service landscape. Public health professionals could prioritize establishing a regional network to bring together all partners working on smoking cessation support services. Such an approach is possible, as seen in England where local governments work on helping smokers to quit (by e.g. increasing quit attempts) next to the goal of promoting smoke-free environments [26]. This report shows, among other things, that nearly 80% of the local authorities had established regional or supra-local networks and partnerships. England’s approach goes beyond merely establishing a network, considering local authorities also deliver a wide range of community stop smoking services. However long-term national funding and structural support are needed for this approach. We recommend that if local governments are unsure of their role, they start by mapping the smoking cessation support services available in the region. This information can then be shared with relevant stakeholders (such as general practitioners). In this way, the public health professionals can build a regional network focused on smoking cessation.

Another factor that could help is that more collaboration could be encouraged and facilitated by the national government, both between public health departments and between national organisations and public health departments. Hendriks et al., [27] for example, highlighted that public health professionals experience less opportunities due to differences in department cultures, which made it difficult for them to work across departments. Public health professionals should more often share knowledge outside their own department to make other departments aware of their influence on tobacco control and the benefits in their interest. For example, a good smoke-free environment with smoke-free entrances ensures a cleaner environment and less visibility of smoking, which could be a benefit to the public space department. This requires additional training in integrated policymaking or, as it is called Health in and for All Policies [28].

### Strength and limitations

A strength of our research is that we obtained interview data from all but one public health department in the Netherlands, thereby obtaining data saturation and near-optimal representativeness of the data. Our findings are therefore representative for all public health regions in the Netherlands. Moreover, this data may be helpful for other countries. While health systems vary internationally, this research specifically highlights challenges faced by countries where local governments are unable to enforce local rules, as is common across Europe. Our study, therefore, extends on current research, which was mostly conducted in the United States, where municipalities can enact tobacco control measures independently of national governments. Additionally, we checked the results by conducting a meeting with the interviewed public health professionals (i.e., a member check), which provided insight into the more complex issues that came out of the interviews.

A limitation of this study is that mainly considers the perspective of public health professionals. While it provides a full view into their experiences, there may still exist barriers and facilitators experienced by municipalities that these professionals on a regional level are not aware of. For future research, it would be worthwhile to develop a more focused topic list to explore these issues in greater depth with municipalities across the country. Additionally, the insight from other stakeholders involved, such as local sports clubs or student organisations might give a more comprehensive insight into the implementation of local tobacco control policies. Also, some of the insights we gained cannot directly be translated into practical recommendations for local policymakers, as many of those challenges identified require action at the national level. These findings highlight the need for improved support of local governments and we hope that national governments could learn from these insights.

An additional strength of this study is the application of the COM-B model, initially used to study individual’s behaviour. The COM-B has recently also been adapted to explore the behaviours of policy-makers. Hendriks et al. [9] for instance used it to examine the behaviours of local policy-makers addressing childhood obesity. The COM-B is part of the Behaviour Change Wheel to clarify the necessary organizational aspects to facilitate individual behaviours such as training, legislation, etc. [8, 9]. The combined COM-B of individuals offers valuable insights, highlighting areas where the organization needs improvement of adjustment. Future research could use the COM-B model to identify the key organizational factors necessary to drive meaningful change.

## Conclusion

Municipalities and local public health departments may contribute to tobacco control by creating smoke-free areas and offering services to help people stop smoking. Execution of these tasks is more difficult in the absence of a clear designated task given by the national government regarding tobacco control. Therefore, tobacco control policies remain non-committing, subject to arbitrariness and dependent on individual characteristics of the local professionals responsible for shaping the policy. The national government could set tasks and goals for public health departments on smoke-free environments and smoking cessation support. Additionally, improved collaboration between the national government and regional public health departments and knowledge exchange between public health departments could help address complex issues such as enforcing smoke-free environments, coordinating prioritizing smoking cessation services, and changing social norms.

## Data Availability

The data that support the findings of this study are not openly available due to reasons of sensitivity and are available from the corresponding author upon reasonable request.
